# Epidemiology and outcomes of children with renal failure in the pediatric ward of a tertiary hospital in Cameroon

**DOI:** 10.1186/s12887-017-0955-0

**Published:** 2017-12-06

**Authors:** Marie Patrice Halle, Carine Tsou Lapsap, Esther Barla, Hermine Fouda, Hilaire Djantio, Beatrice Kaptue Moudze, Christophe Adjahoung Akazong, Eugene Belley Priso

**Affiliations:** 1 Faculty of medicine and pharmaceutical sciences, University of Douala, Douala, Cameroon; 2 Department of internal medicine, Douala general hospital Cameroon, PO Box: 4856, Douala, Cameroon; 3 Faculty of medicine and pharmaceutical science, University of Douala, Douala, Cameroon; 4 Department of paediatric and neonatology, Douala general hospital, Douala, Cameroon; 5 Faculty of medicine and biomedical sciences, University of Yaoundé I, Yaoundé, Cameroon; 6 Higher Institute of Health Sciences, Université des Montagnes, Bangangté, Bangangté, Cameroon; 7 Departement of pediatric and neonatology, Douala Laquintinie hospital; Higher Institute of Health Sciences, Université des Montagnes, Bangangté, Cameroon; 8 Department of gynaecology, Douala general hospital Cameroon, Douala, Cameroon

**Keywords:** Epidemiology, Outcome, Renal failure, Pediatric, Cameroon

## Abstract

**Background:**

Pediatric nephrology is challenging in developing countries and data on the burden of kidney disease in children is difficult to estimate due to absence of renal registries. We aimed to describe the epidemiology and outcomes of children with renal failure in Cameroon.

**Methods:**

We retrospectively reviewed 103 medical records of children from 0 to 17 years with renal failure admitted in the Pediatric ward of the Douala General Hospital from 2004 to 2013. Renal failure referred to either acute kidney injury (AKI) or Stage 3–5 chronic kidney disease (CKD). AKI was defined and graded using either the modified RIFLE criteria or the Pediatrics RIFLE criteria, while CKD was graded using the KDIGO criteria. Outcomes of interest were need and access to dialysis and in-hospital mortality. For patients with AKI renal recovery was evaluated at 3 months.

**Results:**

Median age was 84 months (1QR:15–144) with 62.1% males. Frequent clinical symptoms were asthenia, anorexia, 68.8% of participants had anuria. AKI accounted for 84.5% (*n* = 87) and CKD for 15.5% (*n* = 16). Chronic glomerulonephritis (9/16) and urologic malformations (7/16) were the causes of CKD and 81.3% were at stage 5. In the AKI subgroup, 86.2% were in stage F, with acute tubular necrosis (*n* = 50) and pre-renal AKI (*n* = 31) being the most frequent mechanisms. Sepsis, severe malaria, hypovolemia and herbal concoction were the main etiologies. Eight of 14 (57%) patients with CKD, and 27 of 40 (67.5%) with AKI who required dialysis, accessed it. In-hospital mortality was 50.7% for AKI and 50% for CKD. Of the 25 patients in the AKI group with available data at 3 months, renal recovery was complete in 22, partial in one and 2 were dialysis dependent. Factors associated to mortality were young age (*p* = 0.001), presence of a coma (*p* = 0.021), use of herbal concoction (*p* = 0.024) and acute pulmonary edema (*p* = 0.011).

**Conclusion:**

Renal failure is severe and carries a high mortality in hospitalized children in Cameroon. Limited access to dialysis and lack of specialized paediatric nephrology services may explain this dismal picture.

## Background

Pediatric nephrology is very challenging and is not a priority in developing countries contrary to developed one [1]. Renal disease in children is common with increase prevalence of chronic kidney disease (CKD) globally and an annual incidence rate of 8% [2–5]. In addition, acute renal failure, now call acute kidney injury (AKI), is common in children admitted to hospitals, with a pooled incidence estimated at 33.7% [6]. The burden of kidney disease in children in most developing countries including Sub-Saharan Africa (SSA) is unknown and difficult to estimate due to lack of data on pediatric kidney disease and absence of renal registries in general. Few hospital based studies exist and the reported pattern of renal disease in pediatric population is variable [7–13]. In developing countries the major causes of CKD in children are chronic glomerulonephritis, urologic malformations (posterior urethral valves) and CKD of unknown etiology, while for AKI septicemia, diarrhea, malaria, and hemolytic uremic syndrome are the most frequent causes [13–20].

In Africa and in SSA especially, lack of advanced diagnostic infrastructure treatment facilities, and human resources often leads to inaccurate diagnosis and suboptimal treatment of children with renal diseases. A recent meta-analysis on the outcome of AKI in children in SSA reported that most children presented with severe AKI, with high need for dialysis (66% of them) compared to the pooled world need for dialysis in AKI of 11% [6, 21]. The main reasons were late presentation to hospital, the cost of care, the use of clinical criteria for diagnosis, which appear only at an advanced stage of the disease. Consequently, morbidity and mortality is especially high in SSA where access to dialysis is very limited. Dialysis is not available in all services and only 64% of children with need of dialysis could receive the therapy. Outcome of these children is therefore very poor with a mortality rate estimated at 34%, much higher than the world rates of 13.8% [6, 21–24].

In Cameroon a country in SSA, nephrology and hemodialysis was expanded in the last decade but more for adults. Pediatrics nephrology is not a priority and the country count only two pediatric nephrologists. General pediatricians manage children, and when necessary they called adult nephrologists for further workup and treatment. Few data exist on the burden of kidney disease amongst adults in Cameroon [25, 26] but data on pediatric renal diseases are inexistent despite the presence of risk factors. We conducted this study with the aim to report the epidemiological profile and outcome of renal failure among hospitalized children in the main tertiary referral hospital of Cameroon and highlighting the challenges of care. This basic data may help health policy makers to plan measures that can improve the condition of children and prevent the disease.

## Methods

The study was carried out at the pediatric unit of the Douala general hospital in the littoral region of Cameroon. Douala general hospital is the main one of two tertiary hospitals of the country and the referral hospital for all patients with kidney disease in the region of about 3 million inhabitants. The pediatrics unit has a team of seven general pediatricians that provide care to children mostly referred from others hospital; no nephrologist pediatrician is available and children with kidney disease are followed up by general pediatrician and adult nephrologists when necessary. Hemodialysis is the only renal replacement therapy available in the country. Access to care in Cameroon is not free but rather by payment mostly out of pocket for the majority due to lack of health insurance.

We retrospectively reviewed medical records of children from 0 to 17 years with renal failure admitted in the pediatric ward from 2004 to 2013. On admission in that ward, all children routinely have a complete full blood count, malaria test, serum electrolytes, urea and creatinine. In children with renal abnormalities on admission, kidney test are repeated as needed. An abdominal ultrasound is routinely done for all children with renal impairment. Renal failure referred to either AKI or Stage 3–5 CKD. AKI was defined using either the modified RIFLE criteria (2004–2007) as an absolute increase or decrease of serum creatinine of at least 1.5, or estimated glomerular filtration rate (eGFR) of more than 25% from baseline (value on admission), or a reduction in urine output of less than 0.5 ml/kg per hour for more than 6 h [27], or using the Pediatrics RIFLE criteria (2008–2012), as urine output,0.5 ml/kg/h for greater than eight hours and /or an estimated creatinine clearance (eCCl) decrease of at least 25%; If previous eGFR was unavailable a baseline eGFR of 100 ml/min/1.73 m2 was assumed [28].

The diagnosis of CKD was based on the eGFR lower than 60 ml/min/1.73 m^2^, in a patients with either previous abnormal creatinine value and/ or urine abnormalities for more than 3 months, associated with one or more of the following: risk factor for CKD (ex: past history of glomerular disease, urologic malformation) presence of bilateral schrunken kidney, hypocalcemia, hyperphosphoremia. [29]

eGFR was determined with the Schwartz formula, using height and serum creatinine [30, 31]. Data collected were: socio demographic information such as age and gender, clinical (temperature, signs and symptom related to renal failure and primary disease, daily urine flow rate, primary diagnosis, laboratory results (kidney test, full blood count) and outcomes (the need and access to dialysis and in-hospital mortality). For patients with AKI renal recovery (decreased of serum creatinine on admission or increase of e CCl) was evaluated at 3 months.

### Definitions

Total renal recovery was considered when creatinine or eGFR at 3 months returned to normal or to baseline value for those with CKD. Partial recovery when serum creatinine at 3 months decrease or eGFR increase from the baseline value but did not return to normal and no recovery when at 3 months serum creatinine increase or eGFR decreased compared to admission values or if the patient remain on hemodialysis.

The cause of AKI was taken as the major diagnosis leading to AKI in the child. Sepsis was defined as the presence a systemic inflammatory response (fever >38 °C, high white cell count at presentation) an increased C- reactive protein level due to suspected or proven infection (by positive culture or tissue stain) caused by any pathogen or a clinical syndrome associated with a high probability of infection [32]. Severe malaria was defined as the presence of fever with presence of plasmodium falciparum on peripheral blood film associated with one or more organ dysfunction such as hypotension, coma, need of ventilation, hematologic involvement. Diarrhea was the passage of three or more loose stools per day.

Chronic glomerulonephritis was based either on a history of a documented glomerular disease or the presence of a glomerular syndrome on admission (proteinuria and/or hematuria, hypertension with bilateral small kidney, decrease eGFR, in the absence of identifiable secondary causes. Diagnosis of posterior urethral valves was made on a history of documented urology malformation, and/or on ultrasound scan and micturating cystourethrogram record. Anuria was defined as urine output less than 1 ml/kg/day.

### Statistical analysis

Data analysis was done with the aid of a software program statistical package for social science (SPSS) version 20. Descriptive statistics used comprised percentages and mean ± standard deviation (SD) and median (IQR). Logistic regression was used to look for factors associated to death. A *p* value <0.05 was considered statistically significant.

## Results

A total of 103 patients’ records (62% males) were included. The median age was 84 months (IQR: 15–144). The most frequent clinical symptoms were asthenia (97.8%), anorexia (92.3%) oedema (38.8%) and vomiting (37.8%). In total 68.8% (55/103) of participants had anuria. (Table 1).

**Table 1:** Clinical and biological characteristics of the study population

Variables	
Median age (months) (IQR)	84 (15–144)
Male gender n (%)	64 (62.1)
Herbal concoctions n (%)	24 (23.3)
Urine flow rate *n* = 80	
Anuria n (%)	55 (68.8)
Oliguria n (%)	7 (8.8)
Conserved n (%)	18 (17.5)
Urine Dipstick (*n* = 76)	
Proteinuria n (%)	
1+	26 (34.2)
2+	18 (23.7)
3+	17 (22.4)
Hematuria n (%)	57 (75)
Leukocyturia n (%)	31 (40.8)
Clinical n (%)	
Asthenia	91 (88.3)
Anorexia	85 (85.2)
Pallor	88 (85.4)
Edema	40 (38.8)
Vomiting	39 (37.9)
Dyspnoea	28 (27.2)
Diarrhoea	24 (23.3)
Coma	19 (18.4)
Obnubilation/drowsiness	16 (15.5)
Agitation	11(10.7)
Biology (Mean ± ET)	
Urea	2.14 ± 1.38
Creatinine	77.36 ± 82.01
Hemoglobin	7.68 ± 2.42
Natremia	132.32 ± 16.29
Kaliemia	4.96 ± 1.46

AKI accounted for 84.5% (*n* = 87) and 86.2% (75/87) were in stage F, with acute tubular necrosis 57.5% (*n* = 50/87) and pre-renal AKI 35.6% (*n* = 31/87) being the most frequent mechanisms (Table 2). Main etiologies of AKI were sepsis 57.5% (50/87), severe malaria 21.8% (19/87), hypovolemia 16.1% (14/87) and herbal concoctions 6.9% (6/87) (Table 3).

**Table 2:** Type, stage and mechanism of renal failure amongst participants

Variables	
Type of renal failure n (%)	
AKI	87 (84.5)
CKD	16 (15.5)
Stages of AKI n (%)	
R	0 (0)
I	12 (13.8)
F	75 (86.2)
Stages of CKD n (%)	
3	0 (0)
4	3 (18.8)
5	13 (81.3)
AKI mechanism n (%)	
Acute tubular necrosis	50 (57.5)
Pre-renal	31 (35.6)
Glomerular	4 (4.6)
Vascular	2 (2.3)
CKD mechanism n (%)	
Glomerular	9 (56.2)
Obstructive	7 (43.8)

**Table 3:** Etiologies of AKI

Etiologies	N (%)
Sepsis	50 (57.5)
Severe malaria Hypovolemia	19 (21.8) 14 (16,1)
Diarrhea	8
Hemorrage	3
Heart Failure	3
Herbal concortion	6 (6.9)
Glomerulonephritis	4 (4.6)

CKD accounted for 15.5% (*n* = 16) and Stage 5 was the most frequent (81.3%). (Table 2). Chronic glomerulonephritis (9/16) and urologic malformations (7/16) mainly posterior urethral valves were the causes of CKD (Table 4).

**Table 4:** Etiologies of CKD

Etiologies	N (%)
Posterior Urethral valves	7 (43.8)
Chronic Glomerulo nephritis	9 (56.2)

**Table 5:** Outcome

Variables	Total *N* = 103	AKI *N* = 87	CKD *N* = 16	*P*
Median hospital stay (IQ)	6 (2–12)	6 (1–12)	6.5 (2.25–11.25)	0.76
Dialysis indication	54 (56.8)	40 (45.9)	14 (87.5)	0.01
Dialysis done	35 (64.8)	27 (67.5)	8 (57.1)	0.49
Reasons for non dialysis				
Lack of adapted equipment	11 (57.9)	8 (57.1)	3 (50)	1
Lack of funds	2 (10.5)	1 (7.1)	1 (16.7)	0.52
Uncontrolled immunodepression (HIV)	1 (5.3)	0 (0)	1(16.7)	0.30
Early death	5 (26.3)	5 (35.7)	1 (16.7)	0.61
Renal recovery at 3 months(*n* = 25)				
Complete		22 (88)		
Partial		1 (4)		
No recovery		2 (8)		
Renal recovery unknown		62 (71.2)		
Lost to follow		18 (20.6)		
Death		44 (50.6)		
CKD outcome at 3 months				
On dialysis			2 (12.5)	
Lost to follow up			6 (37.5)	
Death			8 (50)	
Mortality	52 (50.5)	44 (50.6)	8 (50)	0.96
Median delay (days) (IQ)	2 (1–6)	2 (1–6)	3 (2–5.5)	0.23

**Table 6:** Factors associated to death in the study population (multivariate analysis)

Variables	aOR	CI: 95%	*p*
Age ≤ 96 months	6.18	2.20–17.34	0.001
Herbal concoctions	4.29	1.21–15.19	0.024
Coma	6.79	1.33–34.58	0.021
Acute pulmonary oedema	4.46	1.40–14.20	0.011

Eight of 14 (57.1%) patients with CKD, and 27 of 40 (67.5%) with AKI who required dialysis, accessed it. Reason for non-dialysis were lack of adapted equipment (57.9%), early death (26.3%), lack of finances (10.5%) and severe immunodepression (5.3%). Loss to follow up in CKD group was 37.5% (6/16) and 20.7% (18/87) in AKI. Of the 25 patients in the AKI group with available data at 3 months, renal recovery was complete in 22 (88%), partial in 1 (4%) and 2 (8%) were dialysis dependent. In- hospital mortality was 50.7% for AKI and 50% for CKD (Table 5). Factors associated to mortality were age < 96 months, (*p* = 0.001), the presence of a coma (*p* = 0.021), the use of herbal concoction (*p* = 0.024) and the presence of acute pulmonary oedema (*p* = 0.011). (Table 6).

## Discussion

This study describe for the first time the epidemiology and outcomes of renal diseases in hospitalized children in the main tertiary referral hospital in Cameroon. The results show that children were mainly males, AKI accounted for the majority. Patients presented with severe disease. Chronic glomerulonephritis and urologic malformations were the causes of CKD while for AKI acute tubular necrosis and pre-renal AKI were the most frequent mechanism, with sepsis, severe malaria and herbal concoctions being the main etiologies. Almost half of the patients with need of dialysis did not access to the treatment mainly due to lack of adapted material, early death and financial constraint. Half of the patients died in the hospital, more than 1/4 of patients were lost to follow up. Renal recovery at 3 months in the AKI group was complete for the majority and two patients were dialysis dependent.

In this study, male patients were predominant. This is in accordance with other studies from developing countries and could be explained by the increased susceptibility of boys to renal disease and probably by some discrimination against women as it has been reported in SSA [18, 19, 21, 33, 34, 35]. Globally patients presented with severe renal insufficiency independently of the type. This presentation with severe disease has been reported in others studies in low-income countries in general and in SSA in particular [20, 21, 34, 36]. Possible reasons are: lack of early diagnosis, inadequate management of potential treatable risk factors, the silent evolution of kidney disease making that patients look for hospital only when the disease is manifested, and especially financial constraints that is a major concern in SSA where the majority of people are poor, and medical care is out of pocket payment [37].

The prevalence of CKD in this study was 15.5% amongst hospitalized children. This high figure was reported in studies in developing countries such as Iran (14.9%), in Jordan (17.3%) Nigeria (20.3%) [10, 38, 39]. In contrast, El Tigani et al. in Soudan [40] found a lower prevalence of 4%. Chronic glomerulonephritis and urologic malformations mostly posterior urethral valves were the mains etiologies of chronic renal failure in the present study. Ibasdin et al. in Nigeria [41] had similar findings. In most developing countries, chronic glomerulonephritis was the main etiology of CKD, with prevalence ranging from 30 to 60%. This could be due to the high prevalence of bacterial and parasitic infections that commonly affect the kidneys in developing countries [18, 19, 42, 43]. In contrast, urologic malformation was the main etiology in most western countries and the 3rd leading cause in Soudan [5, 40, 44]. Posterior urethral valve, a surgically treatable condition was the main urologic malformation responsible for chronic renal failure, raising the problem of late diagnosis, inadequate management of these children and inability to afford for the surgery. In this study, AKI was mainly due to sepsis, severe malaria and hypovolemia. This is in accordance with reported finding in SSA [20, 21, 34].

It is well known that access to renal replacement therapy (RRT) is very limited in SSA with a huge gap between those who required and received [45, 46]. In the present study an average of 62.2% of children who required dialysis, accessed it. This is in the range of the mean percent access to dialysis for children in SSA [21, 47]. Main reasons for non-dialysis in this study were lack of adapted dialysis material, early death due to late presentation, and financial restriction. Similarly Olowu et al. identified the same factors amongst children with renal failure in Nigeria (24).

Mortality rate and loss of follow up of children in the present study was high a situation already known in SSA [21, 32, 47, 48]. Almost half of the children died during hospitalization independently of the renal failure type. Compared to the pooled mortality rate of children in the world in general and in SSA in particular, our mortality for AKI was higher but in contrary lower for CKD [6, 21, 47]. This high mortality rate could be explained by many factors such as: the severity of the renal disease at presentation in the hospital, the impact of the underlying disease, the lack of adequate infrastructure, the financial constraints especially for those with end stage renal disease requiring hemodialysis. It is known that the cost of hemodialysis is unaffordable for most families in SSA [18, 24, 37, 49–52]. Attention should be paid to all these factors and the development of preventive nephrology in SSA is very important. This will reduce the morbidity and mortality of renal failure amongst children.

Our study has some limitations: the retrospective nature in which accuracy of data collection can be doubted. All the shortcoming of such a study design such as the absence standardization in the assessment of variables and the issue of missing data or cases. Also because of lack of diagnosis facilities (renal biopsies, genetic test), some disease may have underestimated in this study. Despite these limitations this study report for the first time the epidemiology and outcome of renal failure amongst children in Cameroon. This basic data give information that could help health planners in future to improve the outcome of children in our setting and to prevent the disease.

## Conclusion

In conclusion, our data revealed that renal failure, especially AKI is common amongst children in Cameroon and they presented to the hospital with severe disease. Access to dialysis was limited by various factors and mortality rate was very high. This study also showed the challenges of the care of children with renal failure in a resource limited setting, where pediatrics nephrologists are almost inexistent. Implementation of health insurance, education of the population, and training of pediatric nephrologist are measure that could improve the outcome of these patients. More importantly is the prevention and treatment of primary disease.

## Abbreviation

AKI: Acut Kidney Injury
CKD: Chronic Kidney Disease
eGFR: estimated Glomerular Filtration Rate
SSA: Sub Saharan Africa
